# A Multi-Sensor for Direct and Simultaneous Monitoring of Changes in the Contents of Four Ionic Components

**DOI:** 10.3390/molecules30051118

**Published:** 2025-02-28

**Authors:** Barbara Niemiec, Robert Piech, Beata Paczosa-Bator

**Affiliations:** Faculty of Materials Science and Ceramics, AGH University of Krakow, Mickiewicza 30, PL-30059 Krakow, Poland; bniemiec@agh.edu.pl (B.N.); rpiech@agh.edu.pl (R.P.)

**Keywords:** potentiometric multi-sensor, ion-selective electrode, potentiometric sensor, paste electrode, carbon black, ruthenium dioxide hydrate, ion analysis

## Abstract

This paper presents the application of a multi-sensor with a renewable surface based on a carbon black paste modified with ruthenium dioxide hydrate for monitoring the concentration changes of four ionic compounds (nitrate, ammonium, sodium, and calcium). By combining these into one sensor body, analyses can be performed simultaneously, based on a single standard curve, on a small number of available samples. The multi-sensor electrodes were characterized by determining both their electrical parameters, using methods such as chronopotentiometry and electrochemical impedance spectroscopy, and analytical parameters, through a series of potentiometric tests. The electrodes were characterized by high electric charge capacities ranging from 80 µF for the sodium electrode to 257 µF for the nitrate electrode. The tested electrodes showed calibration curve slopes of −51.1 mV/dec for the nitrate electrode, 59.3 mV/dec for the ammonium electrode, 57.0 mV/dec for the sodium electrode, and 26.0 mV/dec for the calcium electrode. The multi-sensor parameters allow for free determination of ions of biological significance in river water samples, soil samples, and plant substrates. The multi-sensor presented in this work can be successfully used to analyze water or plant substrates at home or among commercial crops.

## 1. Introduction

Ion-selective electrodes (ISEs) are the chemical sensors with the longest history. They are characterized by several advantages, such as simple construction, low preparation costs, high sensitivity and selectivity, and no need for sample preparation. Ion-selective electrodes are widely used in clinical, environmental, and industrial analysis. In the group of potentiometric sensors, classic ion-selective electrodes are still often used due to their response stability. Nevertheless, they have some disadvantages related to having an internal solution: among others, ease of destruction, the need to replenish the internal solution, the need to keep the electrode in a vertical position, and leakage of the internal solution into the sample. The disadvantages mentioned above encourage the development of alternative methods. Ion-selective paste electrodes may be the answer to the problems that occur with classic electrodes because they do not have an internal solution in their structure. In addition, they are cheap and easily renewable, as preparation of a paste electrode is easy and takes only a few minutes. They also have advantages compared to solid-contact electrodes: they reduce the consumption of the harmful solvents needed to prepare the intermediate layer, and there is no need to use complicated methods to prepare the paste.

Adams was the first to use paste electrodes in electroanalysis in 1958 [1]. Mersarci and Damen used paste electrodes in potentiometry [2]. The construction of paste electrodes involves filling a cavity in an electrode body with paste. The prepared, leveled surface of the paste forms a modified electrode substrate. Classically, carbon paste is a mixture of graphite powder, a non-conductive liquid binder, and an electroactive material. Currently, other carbon nanomaterials, such as graphene, are also introduced into pastes [3]. Paste electrodes are characterized by the easy renewability of the paste surface: just squeeze out part of the paste and level its surface again. In this work, classic graphite powder was replaced with carbon black, which was successfully used as an intermediate layer in solid-contact electrodes [4].

This work proposed the construction of a potentiometric multi-sensor for the simultaneous determination of nitrate, ammonium, sodium, and calcium ions. The choice of these ions was dictated by their importance in environmental samples such as soil solutions and water. The multi-sensor consisted of four paste electrodes with a paste based on carbon black (CBP) modified with ruthenium dioxide hydrate. Ruthenium dioxide has been successfully used as the intermediate layer in solid-contact electrodes and is characterized by excellent electrical properties [5,6,7].

The potentiometric multi-sensor allows for simultaneous determination of four ions of biological significance based on a single calibration curve. In comparison to other commonly used methods such as chromatography or spectrophotometry, analysis using a multi-sensor is much cheaper and does not require complicated preparation of samples for analysis.

There are other works in the literature reporting potentiometric multi-sensors for medical and environmental applications. However, the selection of ions for our four-channel sensor is new. Previous works have dealt with sensors sensitive to sodium, potassium, calcium, and magnesium ions, as in the work [8]; nitrate and potassium, as in the work [9]; Na^+^, K^+^, Ca^2+^, Mg^2+^, Cl^−^, and SCN^−^ ions and pH, as in the work [10]; or K^+^, Na^+^, and Ca^2+^ ions, as in the work [11]. None of the previously reported solutions mention construction based on paste electrodes.

An area that may particularly benefit from the development of ion-selective paste electrodes is agriculture. Improving the growth and yield of plants requires strict control of their cultivation conditions. In the context of this work, the aspect of the contents of minerals in soil solutions deserves special attention, with an emphasis on nitrate anions and sodium, calcium, and ammonium cations.

Nitrogen is the main element of the atmosphere of the Earth and an essential ingredient for both terrestrial and marine organisms [12]. Nitrogen deposited on land comes in two main forms: nitrate and ammonium. Ammonium is introduced into the environment mainly by the activities of the agricultural industry (released from fertilizers and animal and human excrement) [13], while nitrates are introduced by both the agricultural sector (released from fertilizers) and the energy sector (combustion of fossil fuels) [14]. Plants need substantial amounts of nitrogen. This macro-element is necessary for the synthesis of, among others, amino acids, proteins, nucleic acids, and chlorophyll. Most plants absorb nitrogen through their root systems in the form of nitrate anions or ammonium cations. The preferential assimilation of one of the forms of nitrogen depends on species conditions and environmental factors. If the concentration of nitrogen compounds is too low, the soil should be fertilized.

Although the presence of sodium is not necessary for the proper growth of most plants, it may be beneficial in conditions of potassium deficiency [15]. This functional nutrient can replace potassium in some metabolic and osmoregulatory functions. However, it is important to prevent excessive accumulation of sodium in soil because above a certain level it is toxic to plants. Excessive sodium accumulation in plant cells inhibits protein synthesis, enzymatic reactions, and the photosynthesis process [16]. Therefore, it is worth controlling both the lower and upper limits of the content of this mineral, but the upper limit is the most important.

Calcium is an ingredient necessary for proper plant growth. It plays a structural role in plant cell walls and membranes, counteracts anions in vacuoles, and is an intracellular transmitter in the cytosol [17].

The ions selected in this work do not only affect the development of plants. Fertilizers used in agriculture can be washed out of the soil and can reach surface waters, such as rivers or reservoirs. As a result, the contents of biophilic ions in water can increase in agricultural areas, affecting aquatic ecosystems. Controlling the contents of biophilic ions in water can play a key role in environmental protection. In addition, river water is often used as a source of drinking water. Excesses or deficiencies of certain ions may have serious health consequences, such as nitrate or calcium ions, while others are almost neutral to health but affect, for example, the taste of drinking water, such as sodium ions. This means that the developed electrodes could potentially be used not only in agriculture but also to evaluate the quality of water.

## 2. Results and Discussion

### 2.1. Electrical Parameters of Multi-Sensor

The tested multi-sensor was characterized using two electrochemical techniques, electrochemical impedance spectroscopy and chronopotentiometry, to determine the electrical characteristics of the electrodes used.

**Figure 1:**
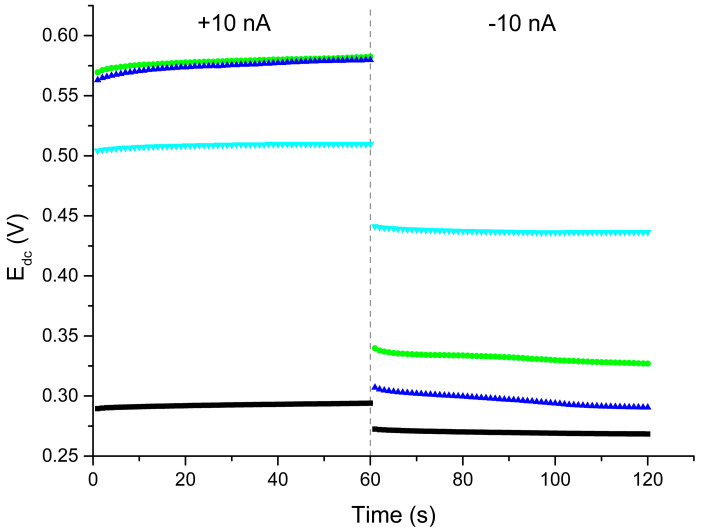
Chronopotentiograms obtained for the CBP-RuO_2_·xH_2_O/NO_3_^−^-ISM (black), CBP-RuO_2_·xH_2_O/NH_4_^+^-ISM (green), CBP-RuO_2_·xH_2_O/Na^+^-ISM (blue), and CBP-RuO_2_·xH_2_O/Ca^2+^-ISM (cyan) electrodes at a current value of 10 nA.

The chronopotentiometry method described by Bobacka [18] allows one to easily determine key electrical parameters (such as potential drift, resistance, and electric capacity) to characterize the potential stability of a potentiometric signal. During the test, a current of 10 nA was forced through the system, at which time a change in potential was recorded. After 60 s of measurement, the direction of the current flow was reversed. Based on the recorded chronopotentiograms (Figure 1), the potential drift (expressed as a change in potential over time (∆E_dc_/∆t)), the capacity of the electric charge (calculated as I∙(∆t/∆E_dc_)), and the resistance (equal to the quotient of the potential jump at the moment of the change in the direction of the current flow and current intensity) were calculated. The electrical parameters determined for each electrode included in the multi-sensor are summarized in Table 1. Based on an analysis of the results obtained, the solvent used in the ion-selective membranes clearly affected the electrical parameters of the electrodes. Among all the component electrodes of the multi-sensor, the lowest resistance was observed for the nitrate electrode with a membrane containing o-NPOE as a plasticizer, in line with observations previously described by Ghauri and Thomas [19].

**Figure 2:**
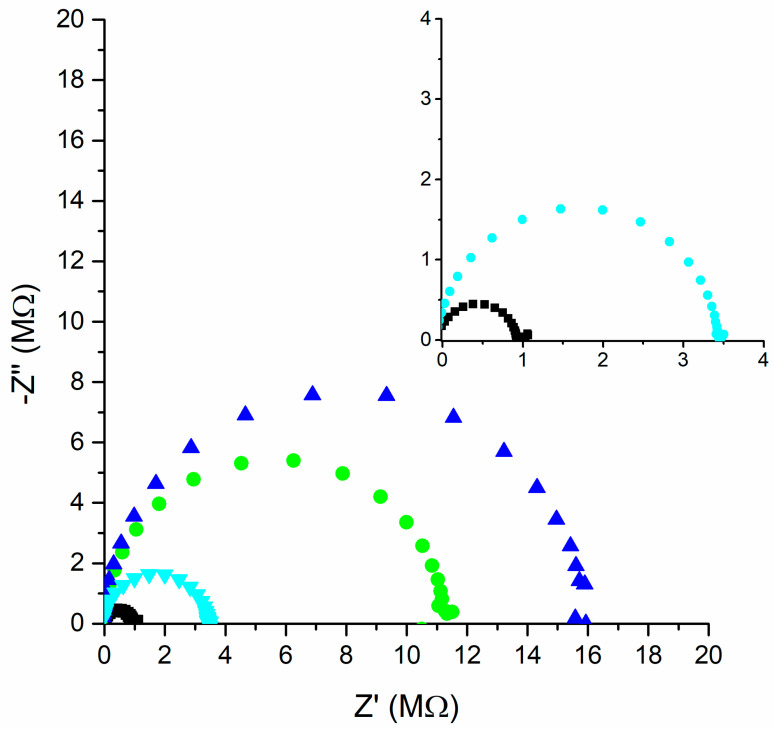
Nyquist plots of the impedance spectra for CBP-RuO_2_·xH_2_O/NO_3_^−^-ISM (black), CBP-RuO_2_·xH_2_O/NH_4_^+^-ISM (green), CBP-RuO_2_·xH_2_O/Na^+^-ISM (blue), and CBP-RuO_2_·xH_2_O/Ca^2+^-ISM (cyan) (frequency range of 100–0.05 Hz with an amplitude of 50 mV).

Electrochemical impedance spectroscopy measurements were performed in the frequency range of 100–0.05 Hz with an amplitude of 50 mV. Nyquist plots, on which the imaginary part of the impedance (Z″) is plotted on the y axis and the real part of the impedance (Z′) is plotted on the x axis, are presented in Figure 2. All impedance spectra show high- to medium-frequency semicircles. A high-frequency semicircle corresponds to the bulk resistance of the ISM in parallel with its geometric capacitance [14]. The bulk resistance is represented by the diameter of the high-frequency semicircle. The resistance values obtained using the electrochemical impedance spectroscopy method are like those obtained by chronopotentiometry, and the relationships between them are maintained. The obtained parameters are collected in Table 2.

### 2.2. Potentiometric Signal Response

**Figure 3:**
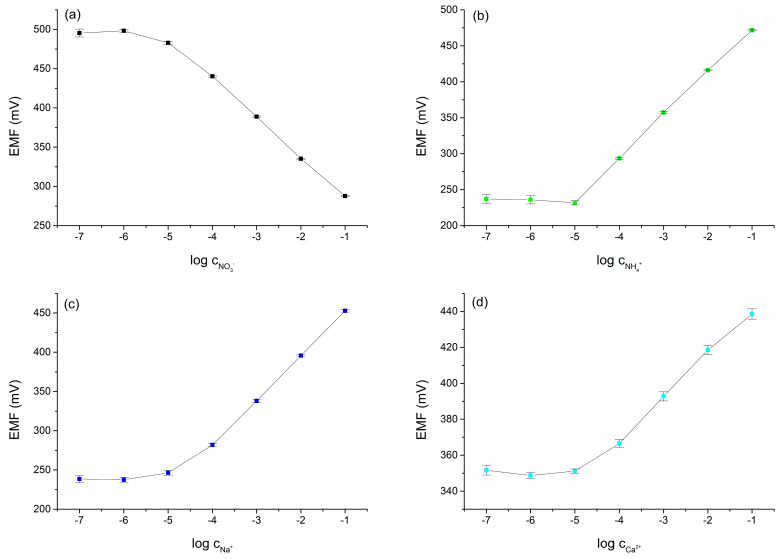
Calibration curves obtained for the CBP-RuO_2_·xH_2_O/NO_3_^−^-ISM (**a**), CBP-RuO_2_·xH_2_O/NH_4_^+^-ISM (**b**), CBP-RuO_2_·xH_2_O/Na^+^-ISM (**c**), and CBP-RuO_2_·xH_2_O/Ca^2+^-ISM (**d**) electrodes from 15 calibrations over 5 days with the electrodes conditioned between measurements.

To investigate the stability of the potentiometric response, fifteen calibrations were performed over 5 days. The calibrations were performed in solutions containing NO_3_^−^, NH_4_^+^, Na^+^, and Ca^2+^ ions with concentrations ranging from 10^−7^ to 10^−1^ M. Between subsequent measurements, the electrodes were conditioned in a mixed solution with 0.01 M concentrations of NH_4_NO_3_, CH_3_COONa, and Ca(CH_3_COO)_2_. The calibration curves obtained in the tests are shown in Figure 3a–d, and the calculated electrode parameters are collected in Table 3.

**Figure 4:**
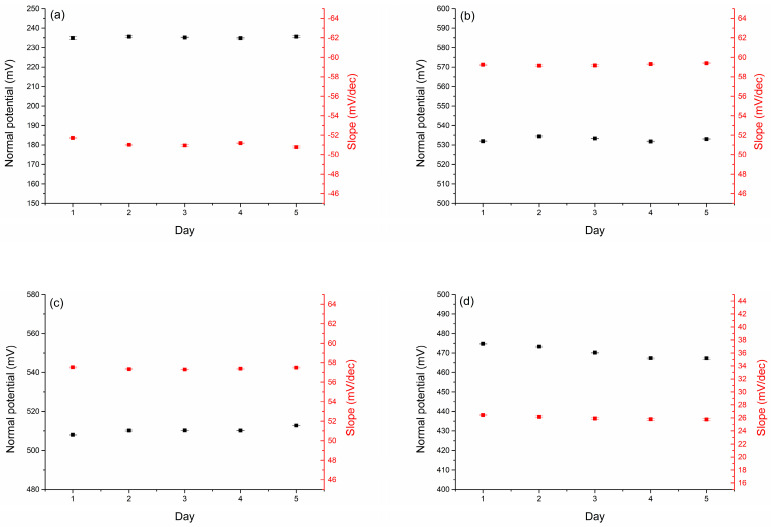
The parameters of the calibration curves (normal potential and slope) determined for the CBP-RuO_2_·xH_2_O/NO_3_^−^-ISM (**a**), CBP-RuO_2_·xH_2_O/NH_4_^+^-ISM (**b**), CBP-RuO_2_·xH_2_O/Na^+^-ISM (**c**), and CBP-RuO_2_·xH_2_O/Ca^2+^-ISM (**d**) electrodes over 5 days. Three subsequent measurements were recorded each day (n = 15).

The slopes of the calibration curve and the normal potential and their changes over 5 days of measurements are shown in Figure 4a–d. Considering the mixed compositions of the conditioning and calibration solutions, favorable analytical parameters were obtained for the multi-sensor and satisfactory repeatability indications were obtained for the electrodes.

**Figure 5:**
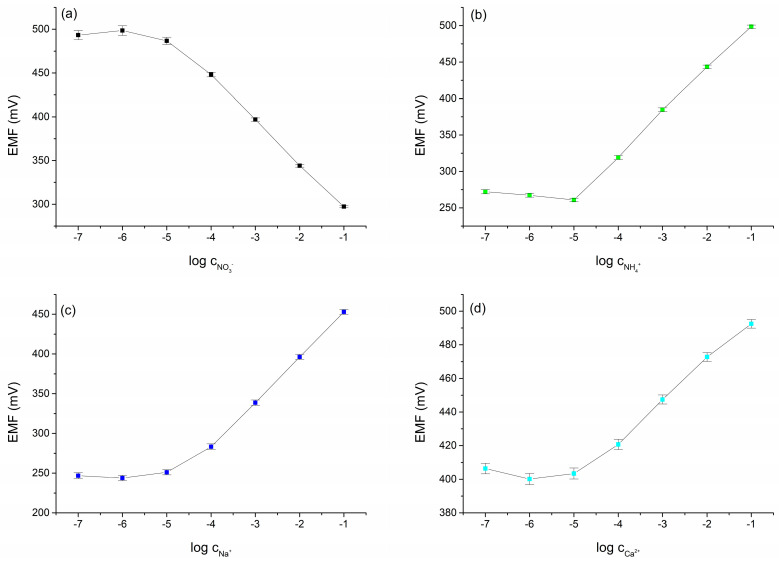
The potentiometric responses of the CBP-RuO_2_·xH_2_O/NO_3_^−^-ISM (**a**), CBP-RuO_2_·xH_2_O/NH_4_^+^-ISM (**b**), CBP-RuO_2_·xH_2_O/ Na^+^-ISM (**c**), and CBP-RuO_2_·xH_2_O/ Ca^2+^-ISM (**d**) electrodes stored dry and conditioned 1 h before measurement (n = 15).

After a series of calibrations between which the electrodes were conditioned, the method of storing the electrodes between measurements was changed to check whether the long conditioning time was necessary to obtain satisfactory repeatability. Electrodes were stored dry and conditioned for only 1 h before measurements were taken. Subsequent calibrations were carried out over 5 days. The results of the subsequent calibrations are shown in Figure 5a–d. The determined analytical parameters of the multi-sensor are summarized in Table 4.

As can be seen in Figure 5d, the way the electrodes were conditioned had an impact on the repeatability of the calcium electrode’s response. However, it still maintained a close-to-Nernstian response. The remaining electrodes, which formed the sensor, maintained appropriate calibration curve parameters until the end of the study.

Another important feature characterizing ion-selective electrodes is potential stability. The stability of the potential was characterized by the potential drift, which was determined as the ratio of the potential change to time. The potential drift value was determined based on the first 20 h of measurement in a solution with the main ion’s concentration at 0.01 M. The following potential drift values were obtained for the tested electrodes: 0.19 mV/h for the nitrate electrode, 0.23 mV/h for the ammonium electrode, 0.13 mV/h for the sodium electrode, and 0.21 mV/h for the calcium electrode. All tested electrodes showed stable long-term responses. The potential drift depended on the ion-selective membrane used.

Table 5 below presents a summary of the parameters of exemplary previously developed ion-selective electrodes for the determination of the ions selected in this work.

### 2.3. Reversibility Test

**Figure 6:**
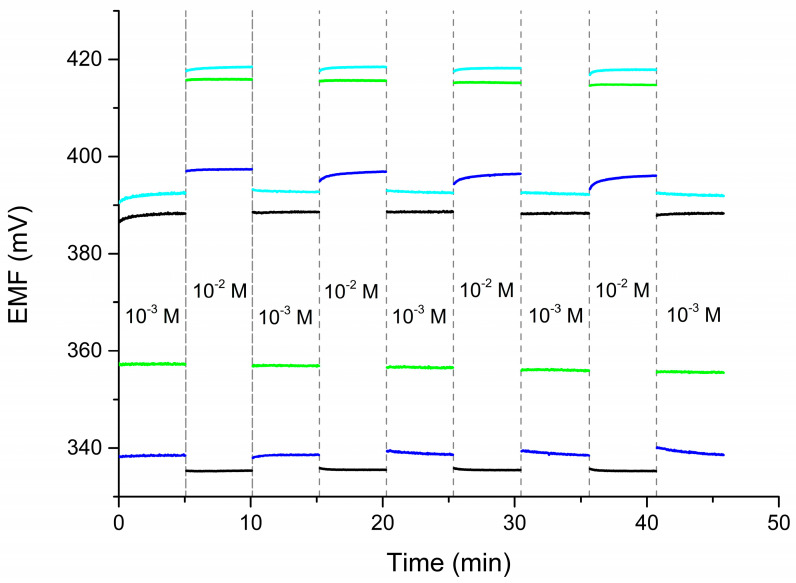
Reversibility of investigated electrodes: CBP-RuO_2_·xH_2_O/NO_3_^−^-ISM (black), CBP-RuO_2_·xH_2_O/NH_4_^+^-ISM (green), CBP-RuO_2_·xH_2_O/Na^+^-ISM (blue), and CBP-RuO_2_·xH_2_O/Ca^2+^-ISM (cyan).

The potential reversibility test involved measuring the potential in rapidly changing solutions with different concentrations. The results are presented in Figure 6. Based on the potential values obtained in solutions with the same concentrations, a standard deviation characterizing the reversibility of the potential could be determined. The standard deviation values for measurements in a solution with a concentration of 10^−2^ M were 0.1 mV for the nitrate electrode, 0.5 mV for the ammonium electrode, 0.6 mV for the sodium electrode, and 0.3 mV for the calcium electrode. For a 10^−3^ M solution, the standard deviations were 0.2 mV for the nitrate electrode, 0.7 mV for the ammonium electrode, 0.09 mV for the sodium electrode, and 0.3 mV for the calcium electrode.

The response times of the multi-sensor electrodes were evaluated according to a method proposed by the IUPAC [23] by measuring the time needed to reach a steady potential (in the range of ±0.5 mV/min) in solutions with concentrations of 10^−2^ and 10^−3^ M. The nitrate electrode reached a steady potential immediately after contact with the 10^−3^ M solution, except during the first cycle, where the response time was 20 s. At a concentration of 10^−2^ M, the response time was up to 5 s. The ammonium electrode showed the fastest response and reached a constant potential immediately after contact with the solution. The response of the sodium electrode stabilized within 15 s in the 10^−3^ M solution and within 60 s in the 10^−2^ M solution. The calcium electrode reached a constant response in the 10^−3^ M solution within a few seconds, except during the first cycle, where it took about 25 s. In the 10^−2^ solution, the response time was up to 15 s.

### 2.4. Light Sensitivity

**Figure 7:**
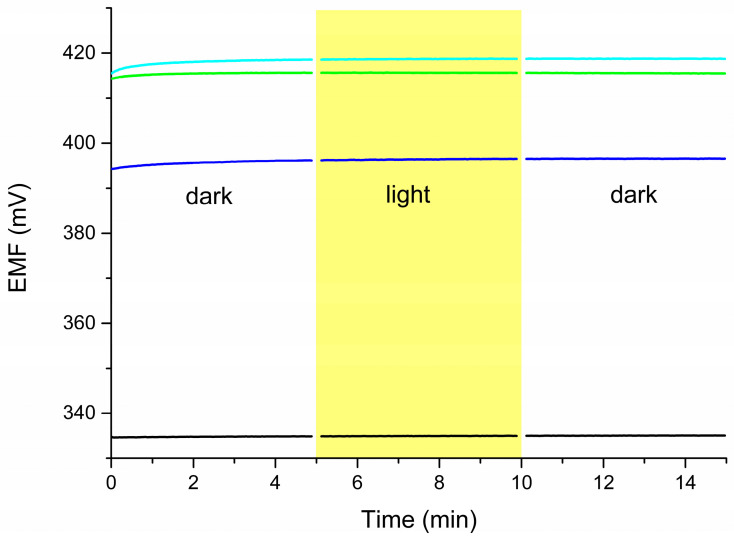
Light sensitivity of CBP-RuO_2_·xH_2_O/NO_3_^−^-ISM (black), CBP-RuO_2_·xH_2_O/NH_4_^+^-ISM (green), CBP-RuO_2_·xH_2_O/Na^+^-ISM (blue), and CBP-RuO_2_·xH_2_O/Ca^2+^-ISM (cyan) electrodes in 0.01 M mixed-ion solution.

The photosensitivity of the electrodes was also investigated. The potential was measured in a dark–light–dark cycle, with each component lasting 5 min (Figure 7). The change in lighting conditions had no effect on the measured potential values. The tested sensor can be used in both daylight and darkness, which expands its potential applications.

### 2.5. pH Sensitivity

**Figure 8:**
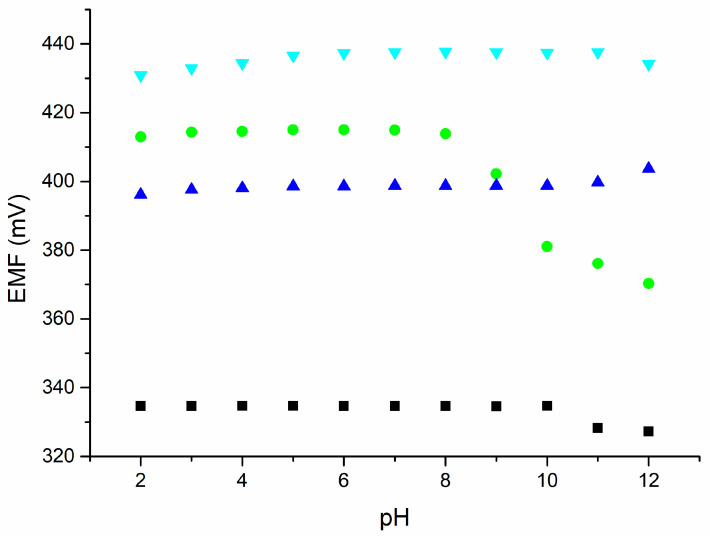
The pH sensitivity of the investigated electrodes. CBP-RuO_2_·xH_2_O/NO_3_^−^-ISM (black), CBP-RuO_2_·xH_2_O/NH_4_^+^-ISM (green), CBP-RuO_2_·xH_2_O/Na^+^-ISM (blue), and CBP-RuO_2_·xH_2_O/Ca^2+^-ISM (cyan) were tested in 0.01 M solutions.

A pH sensitivity test was carried out in a 0.01 M solution of determined ions (Figure 8). Electrode responses were measured in successive solutions with pH values in the range of 2–12. The pH values of the solutions were adapted by adding 3 M KOH or 3 M HCl solutions. The pH range depended on the ion-selective membrane used. The pH ranges for the electrodes were 2–10 for the nitrate electrode, 2–8 for the ammonium electrode, 2–10 for the sodium electrode, and 5–11 for the calcium electrode. The electrodes can be successfully used within these ranges.

### 2.6. Redox Sensitivity

**Figure 9:**
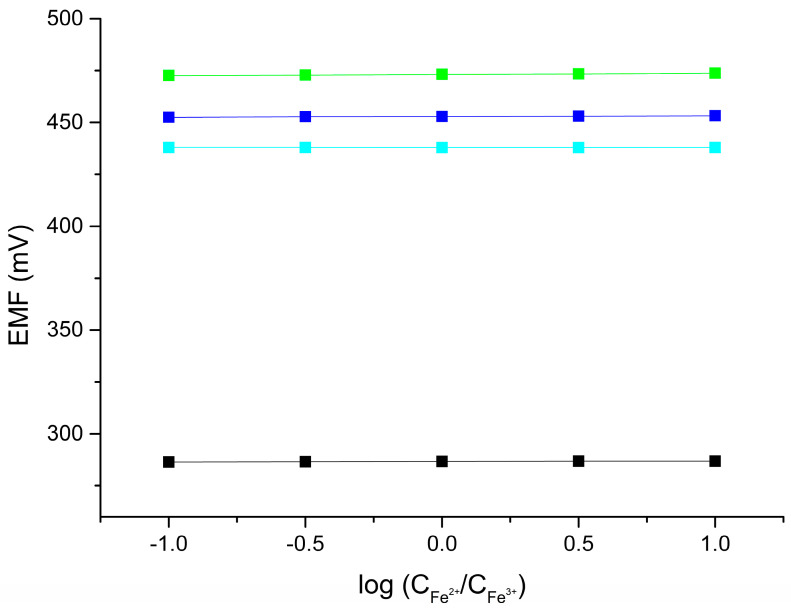
Redox sensitivity of CBP-RuO_2_·xH_2_O/NO_3_^−^-ISM (black), CBP-RuO_2_·xH_2_O/NH_4_^+^-ISM (green), CBP-RuO_2_·xH_2_O/Na^+^-ISM (blue), and CBP-RuO_2_·xH_2_O/Ca^2+^-ISM (cyan) electrodes.

Since electronically conductive materials were used to prepare the multi-sensor base, it was checked whether the ready-to-use sensor showed any redox sensitivity. A test was carried out for all CBP-RuO_2_·xH_2_O/ISM electrodes. The solutions used contained a FeCl_3_ and FeCl_2_ redox couple at a total concentration of 1 mM with the logarithm of the Fe^3+/^Fe^2+^ ratio equal to −1, −0.5, 0, 0.5, and 1 and a constant ionic background of 0.1 M NH_4_NO_3,_ NaCl, or CaCl_2_. As can be seen, no redox sensitivity was observed for the tested multi-sensor (Figure 9).

### 2.7. Water Layer Test and Stability of Potential

**Figure 10:**
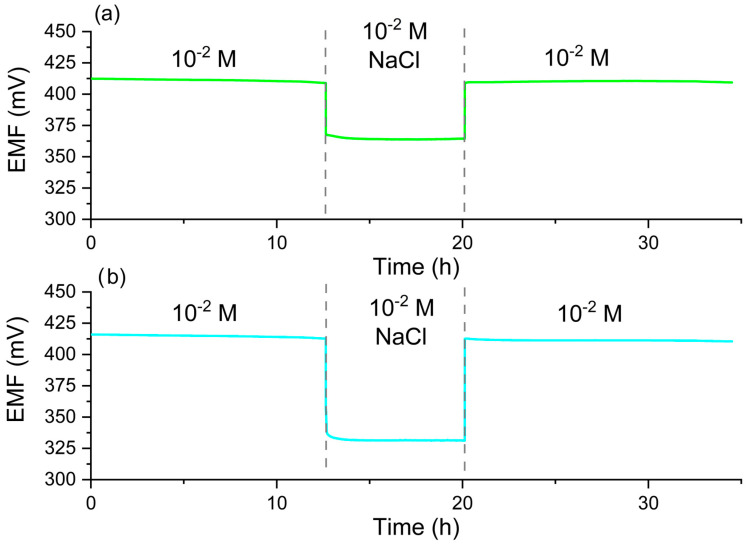
Exemplary water layer tests carried out for the CBP-RuO_2_·xH_2_O/NH_4_^+^-ISM (**a**) and CBP-RuO_2_·xH_2_O/Ca^2+^-ISM (**b**) electrodes in 10^−2^ M solutions of NH_4_NO_3_ and Ca(CH_3_COO)_2_ with 10^−2^ M NaCl as a source of interfering ions.

A water layer test was performed to confirm or deny the presence of a water layer at the interface between the paste and the ion-selective membrane. The presence of a water layer negatively affects potential stability and may cause the membrane to detach from the electrode. The water layer test was performed according to a commonly used procedure proposed by Fibbioli et al. [24]. During the water test, the potential was measured in the main ion solution, then in the interfering ion solution and back in the main ion solution. The presence of a water layer would have been evidenced by a characteristic potential drift during the return to the solution containing the main ions. During the test, no characteristic drift was observed for the tested electrodes. Figure 10 shows examples of the electrode responses during the test for the ammonium and calcium electrodes.

### 2.8. Analytical Application

To show the usefulness of the electrodes in environmental analysis, the contents of nitrate, ammonium, sodium, and calcium ions were determined in river water samples, soil samples, and commercial plant substrates. The soil and plant substrate samples were prepared by mixing 250 g of a sample with 500 mL of deionized water and were separated by filtration after 24 h. The determination of the concentrations of nitrate, ammonium, sodium, and calcium ions was carried out using the standard addition method for three samples of each type. The results of the determinations are presented in Table 6 and Table 7.

The contents of the ions in the soil/plant substrate water extracts were additionally recalculated to reflect their contents in the dry mass of the soil/substrate, considering the moisture contents of the raw samples. The results are presented in Table 8.

## 3. Materials and Methods

### 3.1. Chemicals

The components of the carbon paste were paraffin oil, carbon black (3D Nano, Krakow, Poland), and ruthenium (IV) oxide hydrate (Alfa Aesar, Haverhill, MA, USA).

The ion-selective membranes consisted of the following components: ionophores, including nitrate ionophore V, ammonium ionophore I, sodium ionophore IV, and calcium ionophore I; lipophilic salts, including tridodecylmethylammonium chloride (TDMACl) and potassium tetrakis(4-chlorophenyl)borate (KTpClPB); and plasticizers, including 2-nitrophenyl octyl ether (o-NPOE), Bis(1-butylpentyl) adipate (BBPA), tris(2-ethylhexyl) phosphate (TEHP), Bis(2-ethylhexyl) sebacate (DOS), and poly(vinyl chloride) (PVC). All components were purchased from Sigma-Aldrich (St. Louis, MO, USA).

Ammonium nitrate (NH_4_NO_3_), sodium acetate (CH_3_COONa), calcium acetate monohydrate (Ca(CH_3_COO)_2_·H_2_O), and magnesium acetate tetrahydrate (Mg(CH_3_COO)_2_·4H_2_O) were purchased from CHEMPUR (Piekary Śląskie, Poland). Salt solutions with concentrations from 10^−7^ to 10^−1^ M were used for potentiometry, chronopotentiometry, and electrochemical impedance spectroscopy measurements.

### 3.2. Electrode Preparation

The electrode corpus was made of polyether ether ketone (PEEK) with four through holes drilled in it. Matching stainless-steel rods were placed in the holes, leaving room to fill with paste.

Paste for the electrode with a composition of 0.4 g of carbon black (CB), 0.3 g of paraffin oil, and 0.175 g of hydrous ruthenium dioxide was ground in a mortar until a homogeneous mixture was obtained. The paste was used to fill the four cavities in the paste electrode. The cavity diameter was equal to 2 mm in the 1.7 cm corpus.

The electrodes’ surfaces were covered with 40 µL of an ion-selective membrane (ISM) solution using the drop-casting method. The NO_3_^−^-ISM was prepared according to a slightly modified procedure described by Watts et al. [25]. The NO_3_^−^-ISM solution had the following composition: 1.10% (*w*/*w*) nitrate ionophore V, 0.70% (*w*/*w*) TDMACl, 65.00% (*w*/*w*) o-NPOE, and 33.20% (*w*/*w*) PVC. The NH_4_^+^-ISM was modified by the addition of lipophilic salt. It had the following composition: 1.0% (*w*/*w*) ammonium ionophore I, 0.33% KTpClPB, 66.47% (*w*/*w*) DOS, and 32.2% (*w*/*w*) PVC [19,26]. For the Na^+^-ISM, the composition was 3.0% (*w*/*w*) sodium ionophore IV, 0.3% (*w*/*w*) KTpClPB, 65.6% (*w*/*w*) BBPA, 2.0% (*w*/*w*) TEHP, and 29.1% (*w*/*w*) PVC [27]. The composition of the calcium membrane (Ca^2+^-ISM) was 3.3% (*w*/*w*) calcium ionophore I, 2.1% (*w*/*w*) KTpClPB, 63.7% (*w*/*w*) DOS, and 30.9% (*w*/*w*) PVC [28,29].

**Figure 11:**
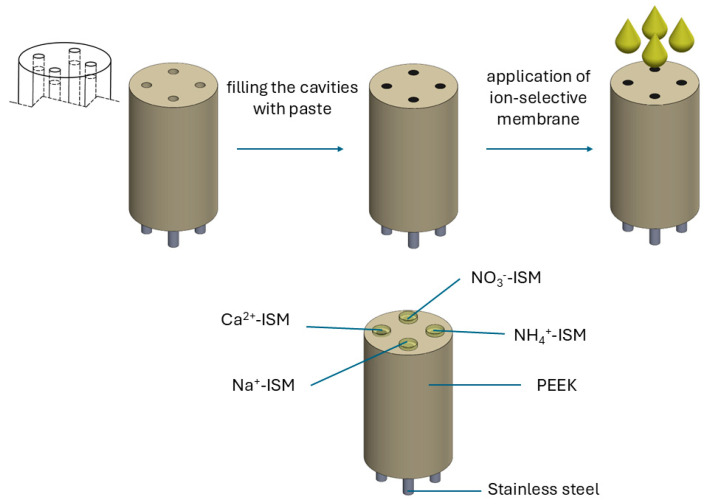
A schematic diagram of the preparation of a potentiometric multi-sensor.

All abovementioned components of each membrane were dissolved in 2 mL of an organic compound with the formula (CH_2_)_4_O (tetrahydrofuran, THF). After applying the membrane solutions to the multi-sensor platform, the THF solvent was evaporated in an air atmosphere under cover for at least 24 h at room temperature. Before the measurements, the multi-sensor covered with a membrane was conditioned in a solution with concentrations of 10^−2^ M of the salts NH_4_NO_3_, CH_3_COONa, and Ca(CH_3_COO)_2_. The steps of preparing the multi-sensor are shown in Figure 11.

### 3.3. Methods

For the chronopotentiometry and electrochemical impedance spectroscopy methods, paste electrodes covered with the ISM were placed into a measuring cell and examined in sequence. Measurements were carried out in a three-electrode system with a single-junction Ag/AgCl electrode and a 3 M KCl reference electrode (the catalogue number from the company ΩMetrohm, Herisau, Switzerland: 6.0733.100), with a carbon glass rod as an auxiliary electrode. The cell was filled with a 0.01 M solution of NH_4_NO_3_, CH_3_COONa, or Ca(CH_3_COO)_2_ as an electrolyte. The measurements were performed using an Autolab General Purpose Electrochemical System (AUT302N.FRA-2-AUTOLAB, Eco Chemie, Utrecht, The Netherlands) that cooperated with NOVA 2.1.4 software.

For the potentiometry method, all prepared paste electrodes in the multi-electrode were connected to a 16-channel mV-meter (Lawson Labs, Inc., Malvern, PA, USA). Measurements were conducted using the same Ag/AgCl electrode as in the measurements of the electrical parameters with a 3 M solution of KCl as a reference electrode in the presence of an additional platinum auxiliary electrode. Potentiometric responses were recorded in standard NH_4_NO_3_, CH_3_COONa, and Ca(CH_3_COO)_2_ solutions with concentrations ranging from 10^−1^ to 10^−7^ M. Calibration solutions contained additional 0.01 M Mg(CH_3_COO)_2_ to ensure constant ionic strength.

## 4. Conclusions

This work shows that paste electrodes based on a carbon black paste modified with ruthenium dioxide hydrate can be successfully used in the construction of a potentiometric multi-sensor for environmental analysis. This multi-sensor enables simultaneous determination of four ions: nitrate, ammonium, sodium, and calcium. The use of a multi-sensor allows for quick analysis of environmental samples. Ion-selective membranes and the compositions of the calibration solutions were carefully selected to enable simultaneous analysis of ions. The simple design of the multi-sensor makes it inexpensive and easy to prepare. The electrodes show a slope close to the Nernst value. The variability in the parameters of the calcium electrode’s calibration curve means that the electrodes require daily calibration. Due to better repeatability of the potentiometric response, longer conditioning of the multi-sensor between measurements is recommended. The multi-sensor responded well throughout the duration of this study. The proposed electrodes can be used both in daylight and in the dark. The proposed sensor is characterized by good metrological parameters and can be used for routine analyses of river water and fertilized plant substrates. This type of sensor can be used to control the levels of fertilization of substrates in both commercial and home crops.

## Figures and Tables

**Table 1:** Electrical parameters of tested carbon black–hydrous ruthenium dioxide paste electrodes.

Carbon Black Paste Sensor CBP-RuO_2_·xH_2_O/:	R_total_ ± SD (kΩ)	∆E_dc_/∆t ± SD (µVs^−1^)	C ± SD (µF)
NO_3_^−^-ISM	1079 ± 1	39.0 ± 0.6	257 ± 4
NH_4_^+^-ISM	12,142 ± 4	113 ± 5	89 ± 4
Na^+^-ISM	13,624 ± 8	125 ± 7	80 ± 5
Ca^2+^-ISM	3427± 5	42.2 ± 0.1	237 ± 4

**Table 2:** The electrical parameters of the electrodes, which were calculated based on equivalent circuits. R_1_—resistance resulting from series connections, R_2_—bulk resistance, CPE—the constant phase element (Y^0^—the initial value for the admittance of the CPE element and an N-parameter showing that if N = 1 the CPE is an ideal capacitance and N = 0.5 means Warburg impedance).

Carbon Black Paste Sensor CBP-RuO_2_·xH_2_O/:	R_1_ (kΩ)	R_2_ (MΩ)	CPE (pS) ^(N)^
NO_3_^−^-ISM	−39.5	0.964	9.68 ^(0.953)^
NH_4_^+^-ISM	−26.7	11.5	8.86 ^(0.976)^
Na^+^-ISM	2.86	15.9	8.21 ^(0.979)^
Ca^2+^-ISM	−54.3	3.48	9.97 ^(0.964)^

**Table 3:** The potentiometric parameters of the investigated carbon black–ruthenium dioxide paste ISEs when conditioned between measurements (n = 15).

Carbon Black Paste Sensor CBP-RuO_2_·xH_2_O/:	Slope ± SD (mV/dec)	Normal Potential ± SD (mV)	Linear Range (M)	LoD ± SD (M)
NO_3_^−^-ISM	−51.1 ± 0.1	235.2 ± 0.5	10^−5^–10^−1^	10^−5.19±0.06^
NH_4_^+^-ISM	59.3 ± 0.1	532.8 ± 0.9	10^−5^–10^−1^	10^−5.09±0.08^
Na^+^-ISM	57.0 ± 0.1	509.6 ± 1.5	10^−4^–10^−1^	10^−4.79±0.02^
Ca^2+^-ISM	26.0 ± 0.3	471 ± 3	10^−4^–10^−1^	10^−4.92±0.04^

**Table 4:** The potentiometric parameters of the investigated carbon black–ruthenium dioxide paste ISEs when stored dry and conditioned 1 h before measurement (n = 15).

Carbon Black Paste Sensor CBP-RuO_2_·xH_2_O/:	Slope ± SD (mV/dec)	Normal Potential ± SD (mV)	Linear Range (M)	LoD ± SD (M)
NO_3_^−^-ISM	−50.5 ± 0.1	245.3 ± 0.9	10^−5^–10^−1^	10^−5.10±0.06^
NH_4_^+^-ISM	60.0 ± 0.1	561 ± 3	10^−5^–10^−1^	10^−4.98±0.03^
Na^+^-ISM	56.6 ± 0.2	509 ± 3	10^−4^–10^−1^	10^−4.75±0.02^
Ca^2+^-ISM	26.0 ± 0.4	525 ± 2	10^−4^–10^−1^	10^−5.14±0.03^

**Table 5:** The parameters of other reported electrochemical sensors for determining the ions selected in this work.

Work	Ion	Linear Range (M)	Limit of Detection (M)	Sensitivity (mV/dec)
[20]	NH_4_^+^	10^−5^–10^−1^	4.1 × 10^−6^	58.1
[21]	NH_4_^+^	10^−5^–10^−1^	2.82 × 10^−5^	51.7
	NO_3_^−^	10^−5^–10^−1^	2.06 × 10^−5^	−54.8
[22]	NO_3_^−^	10^−4.3^–10^−1^	3 × 10^−5^	−57.9
[4]	Na^+^	10^−4^–10^−1^	10^−5^	42.7 ± 0.2
	Ca^2+^	10^−4^–10^−1^	10^−5^	28.7 ± 0.2
[3]	Na^+^	10^−7^–10^−1^	-	56 ± 1
	Ca^2+^	10^−8^–10^−1^	-	29 ± 1

**Table 6:** Determination of nitrates, ammonium, sodium, and calcium in various river water samples by the standard addition method.

Sample	Nitrate Found by NO_3_^−^-ISE, mgL^−1^	Ammonium Found by NH_4_^+^-ISE, mgL^−1^	Sodium Found by Na^+^-ISE, mgL^−1^	Calcium Found by Ca^2+^-ISE, mgL^−1^
Rudawa river water	15.15 ± 0.11	0.73 ± 0.03	15.5 ± 0.4	82.75 ± 1.7
Dunajec river water	4.78 ± 0.03	0.56 ± 0.02	9.41 ± 0.09	34.1 ± 0.8
Biała river water	5.74 ± 0.09	0.83 ± 0.03	18.5 ± 0.5	64.5 ± 1.3

**Table 7:** Determination of nitrates, ammonium, sodium, and calcium in various soil samples (water extracts) by the standard addition method.

Sample	Nitrate Found by NO_3_^−^-ISE, mgL^−1^	Ammonium Found by NH_4_^+^-ISE, mgL^−1^	Sodium Found by Na^+^-ISE, mgL^−1^	Calcium Found by Ca^2+^-ISE, mgL^−1^
Substrate for green plants	850 ± 30	20.0 ± 0.6	41.5 ± 0.5	267 ± 4
Substrate for flowering plants	1020 ± 20	9.1 ± 0.2	30.3 ± 0.6	268 ± 2
Soil	<LoD	0.72 ± 0.07	2.6 ± 0.2	34.3 ± 0.4

**Table 8:** The contents of nitrate, ammonium, sodium, and calcium ions in the plant substrates and soil, calculated on a dry-weight basis for the samples.

Sample	Nitrate Found by NO_3_^−^-ISE, mg/g	Ammonium Found by NH_4_^+^-ISE, mg/g	Sodium Found by Na^+^-ISE, mg/g	Calcium Found by Ca^2+^-ISE, mg/g
Substrate for green plants	4.2 ± 0.1	0.099 ± 0.002	0.206 ± 0.002	1.33 ± 0.02
Substrate for flowering plants	6.7 ± 0.2	0.059 ± 0.001	0.197 ± 0.004	2.39 ± 0.02
Soil	<LoD	0.0019 ± 0.0002	0.0067 ± 0.0006	0.088 ± 0.001

## Data Availability

The original contributions presented in this study are included in the article. Further inquiries can be directed to the corresponding author(s).
